# An introductory review of robotically assisted surgical systems

**DOI:** 10.1002/rcs.2409

**Published:** 2022-05-04

**Authors:** Francesco Cepolina, Roberto P. Razzoli

**Affiliations:** ^1^ Department of Mechanical Engineering, Energy, Production, Transport and Mathematical Models (DIME) University of Genoa Genova Italy

**Keywords:** haptic feedback, laparoscopy, miniature instruments, minimally invasive surgery, remote surgery, robot surgery

## Abstract

**Purpose:**

The scope of the work is to present the state of the art of robotically assisted surgical systems and to give a general idea about how technology can help today and tomorrow robotic surgery. The road to innovation passes through research and on field trials; for this reason, not only commercial surgery robots, but also innovative prototype robots, proposed by the Academic world, are presented.

**Design/methodology/approach:**

Following a short introduction, robotically assisted surgery systems are introduced discussing their architectures and main peculiarities. A further section is dedicated to the key enabling technologies that will make possible to improve current systems and that will lead to a new generation of surgical robotic systems able to meet the patient's needs and facilitate the surgeon's task. Finally, brief concluding comments are given.

**Findings:**

The idea of using robots for surgery was born many years ago and in a short time a market demand was created. Today the market is very dynamic, and several new products are updated and created for the execution of both traditional and new procedures. The article provides a guide for the reader who has an interest in this area.

**Originality/value:**

This paper provides an insight into the commercial robotic surgical systems and a look on research prototypes from academic and industrial worlds.

## 1 INTRODUCTION

In the last decades robots were slowly going outside the industry to serve more closely humans. Today service robots are already working on different fields. Robots are playing an increasingly important role in assisting surgeons to perform a wide range of operations, mainly minimally invasive surgery (MIS).^1^, ^2^ In fact, after a period of scepticism, thanks to the development of new technologies, robotic systems are more reliable, and many patients have fearlessly opted for robotic surgeries. Globally, it is estimated that about 3% of surgeries are performed robotically, offering patients the benefits of MIS, fewer complications, shorter stay in hospital, and faster return to normal activities. In the pandemic period, the role of robots in telemedicine is even more important. Hospitals, by nature, are places where there is a high probability of contacts and infections; the possibility to assist a patient, while keeping the social distancing, offers additional safety for all the medical stuff and the patient, as well.

Robotic surgery needs, in general, limited displacements, high accuracy, low size, and reduced but sufficient forces.^3^ Despite surgical robots have the aim to make surgeons tasks, they still need to leave space to anthropocentric approach. Also, it is not necessary they mimic the same surgeon gestures to achieve the same task. Tailors sew using hands; the sewing machine replicates the same task optimising the whole process in a different way.

Robotically Assisted Surgical Systems (RASS) allow MIS using highly dexterous instrumentation that enables smaller and less traumatic access into the body of the patient.^4^, ^5^ This allows for faster healing so reducing the hospitalisation time and thereby the costs per patient.

The use of robot arms for positioning and holding the surgical tools relieves surgical assistants from physical holding tasks while the trust in an enhanced positioning and working accuracy leads to surgeons' mental relief.

Moreover, RASS have the potential to increase safety for clinicians and patients.

Force feedback is an increasingly available feature allowing doctors to provide a more immersive telepresence; force feedback can be successfully implemented, even for low‐cost remote ultrasound scan procedures.^6^, ^7^

The success of a surgical robot on the market can be found in effectiveness, easiness of use, versatility, and cost.

In the last years many companies that produced surgical robots disappeared and in the recent past significant acquisitions of surgical robot manufacturers have taken place.

The market had a growth of 16% from 2019 to 2020. The trend of surgical robots' market is expected to continue growing at a Compound Annual Growth Rate of over 17.5% from 2021 to 2027.^8^ European Union in the last decades sponsored and is sponsoring the born of new surgical robots. This business needs, generally, much research, money, and time to certify the surgical procedures. Today there is already a complex cloud of patents related to robotic surgery; new ideas need to be well ‘shaped’ in order not to fall in an existing patent. Also, early surgery patents, as usual, are protecting general concepts like, for example, the basic concepts related to ‘remote surgery’.

If today the surgical robotic systems are still not widespread due to the technological complexity, a difficult patents situation, regulatory barriers, absence of standards, high costs and the necessary preparation of surgeons for the new procedures, tomorrow the scenario may be different: it is socially and ethically important surgery robotics to be available to a wide public.

After the introduction, a general overview of the state of the art of robotically assisted systems available on the market and developed at the level of research prototypes is given in the second section.

The third section is devoted to the presentation of some of the most important key technologies that will enable the improvement of the market systems and that will be implemented in the new surgical systems generation.

The last section briefly reports on social fallout of RASS and then the conclusion is presented.

## 2 ROBOTICALLY ASSISTED SURGICAL SYSTEMS: STATE OF ART

Robotic surgical systems are briefly presented below. It is a very dynamic market with acquisition negotiations between the reference industries still in progress and with the interest and commitment of researchers to propose new solutions based on current technologies. Therefore, the presentation is not exhaustive.

### 2.1 The Zeus™ system

In 1995 Computer Motion produced the Zeus system, which was certified by the Food and Drugs Administration (FDA) in 2001, by combining two robotic arms capable of holding instruments with an Aesop robot, previously developed by the same Computer Motion. Doctors can perform surgical actions with direct or remote control.^9^ Zeus is equipped with a computer‐assisted remote‐control device that interacts with a robotic arm. To improve the precision of the robotic arm movements during the operation, the Zeus system filters the tremors of the human hand. In 2001 the Zeus system assisted physicians in successfully completing the first “Lindbergh surgery operation” carried out by a team of French surgeons located in New York on a patient in Strasbourg, France. ZEUS was discontinued in 2003, after Computer Motion has been merged with its competitor Intuitive Surgical.

### 2.2 The Da Vinci^®^ equipment

The Da Vinci^®^ surgical system was born in California. After several years of investments on research, the robot has become a product. Surgical robots need to pass strict tests before to be used; normally it takes a long time before surgical procedures are cleared by the USA FDA. Da Vinci^®^ gained the FDA approval in 2011.

Today da Vinci^®^ is a reference in surgical robotics. The da Vinci^®^ surgical robot from Intuitive Surgical is a versatile system that has found acceptance worldwide through various generations of systems in robotic surgery, such as cardiology, laparoscopy, neurosurgery, microsurgery, orthopaedics, and ophthalmology.

As early as 2020, the Da Vinci^®^ boasted more than 5500 installations globally and performed more than seven million surgeries.

Da Vinci^®^ robot is based on the classic master‐slave operating principle; it has four arms and can be governed by two consoles. Three arms can carry surgical tools, one arm holding the camera. In the last model Xi each arm has three DoF and is equipped with the proprietary Endo Wrist which provides supplementary DoF for a human‐like dexterous manipulation.

The two cameras of the master console provide a magnified 3D view of the surgical field. The master console was created with the surgeon's needs in mind; it has adjustable finger loops, adaptable intraocular distance, a padded headrest and arm bars. Instrument motion is made possible through cable‐driven joints at the distal end of the instrument.

Intuitive Surgical^®^ is constantly devoted to innovation; the company publishes several patents each year related to surgical robots and surgical instruments. For example, the patent US2004/0261179A1 proposes some ceiling and floor mounted surgical robot setup arms. Ceiling mount robotic arms give to the surgical stuff more free space close to the patient. Also, the cleaning of the room and the movement of the bed of the patient is simplified.

The Intuitive Surgical^®^ Ecosystem is subdivided in three main areas: from industry‐leading surgical platform innovation to clinical training and customers support. In order to optimise both the clinical and the economic value, it is vital to handle the full ecosystem as a single integrated process.

### 2.3 M7 surgical robot system

SRI's (Stanford Research Institute's) in 1995 formed Intuitive Surgical, Inc. SRI's in 1998 developed the M7 remote surgery robot. M7 has two anthropomorphic robotic arms with seven force‐reflective degrees of freedom. Surgeons can teleoperate using stereo vision. Each arm weights 4.5 kg. The microsurgical system has been demonstrated for suturing lacerations of the cornea. The NASA Extreme Environment Mission Operations (NEEMO) took place in the ocean on a permanent undersea laboratory, Aquarius in Florida. In April 2006, during the 9th NEEMO project, the system M7 has been used to perform a real time abdominal surgery on a patient simulator. In May 2007, during the 12th NEEMO project, M7 performed on a simulated patient in zero gravity environment. In September 2007 M7 Robot did, aboard a NASA C‐9 aircraft, the first surgical demonstration in a zero‐Gravity flight.^10^

### 2.4 Raven IV equipment

The Bionics lab of the University of California, in Santa Cruz, has developed Raven IV surgical robotics system. To reproduce the original dynamics of two surgeons interacting with the surgical site, Raven IV includes four robotic arms and 2 cameras. The two surgeons can teleoperate using two surgical consoles. Like Da Vinci^®^, one or more assistants may physically cooperate with the robot by replacing tools or interacting with tissues.

One of the main tasks of Raven IV is to make possible a collaborative teleoperation made by two surgeons.^11^ Like Da Vinci^®^, each surgical arm is based on a spherical mechanism with a remote centre located at the entry point of the tool into the human body. The design of this surgical system is light and compact. The Raven IV equipment has been successfully tested; two surgeons were able to remotely complete fundamental laparoscopic surgery tasks.

### 2.5 The PMAR needle equipment

PMAR (design and measurements for robotics and automation) lab, from the University of Genoa, proposes a surgical parallel robot called PmarNeedle. The PmarNeedle is a slave robot purposely designed for needlescopic diagnosis, one of the current frontiers of MIS. The dimensions of the robot are small compared to the robots in use for laparoscopy. While the force capabilities of the system are less than those of a generic full scale surgical robotic system, they are enough to allow even conventional laparoscopic procedures. Each parallel robot of the robotic system is composed by an upper mechanism connecting the two arms of the base structure. The two arms have different geometry to guarantee the largest collision‐free workspace; robot dimensions are synthesised on the base of the surgical requirements. The robot kinematic is centred on the keyhole; in case of control error, the geometry does not allow the instrument to damage the skin and ribs close to the keyhole. The kinematic and dynamic models of the robot have been investigated to define the control laws. Simulink‐SimMechanics is the environment chosen for the simulation; virtual sensors have been implemented to monitor the motion of the robot. Simple Proportional Derivative control laws with and without non‐linearity compensation filters have been implemented. A robot mockup has been prototyped.

### 2.6 Mirosurge equipment

The DLR (German Aerospace Centre) has created MiroSurge, a minimally invasive telesurgery system, designed mainly for research. The telesurgery scenario includes a master console as well as a teleoperator, consisting of 3 surgical robots (MIRO). Usually, two robots carry the surgical instruments MICA equipped with miniaturised force/torque sensors, to capture reaction forces with manipulated tissue. A stereo video laparoscope is guided by one or more robots.^12^ Like Da Vinci^®^, the surgeon remotely controls the operation from a console; the surgeon has 3D endoscopic sight, force feedback, and restored hand‐eye‐coordination. Both the stereo video stream and the measured forces are displayed to the surgeon at the master console. Performance characteristics of the MIROs are designed to follow the stabilised beating heart motion.

### 2.7 MSR‐5000 REVO‐I surgical robot system

The South Korean Meere Company exploiting its previous experience in developing minimally invasive surgical systems, in 2015 realized the MSR‐5000 REVO‐I. The REVO‐I system is a master slave system like the Da Vinci^**®**^ system. It consists of an operating cart supporting four arms with 12 DoF that may be endowed with instruments reusable up to 20 times, the double compared to Da Vinci^®^ instruments, and a surgeon control console with High Definition (HD) vision cart incorporating haptic feedback. The REVO control console precisely transfers the surgeon's hand movement to the robotic arms making easy for the surgeon to drive the surgical procedure. Warning signals are sent from the site to the surgeon to make him aware of possible problems and unexpected happenings. This surgical robot system has received the Korean Food and Drug Administration approval in August 2017.

Meere company also developed RevoSim a virtual reality (VR) training system, through which novice robotic surgeons can improve their psychomotor skills and instrument handling and gain the confidence required to perform robotic surgery.

### 2.8 Senhance surgical robotic system (ALF‐X)

In February 2010, SOFAR SpA, in collaboration with the Faculty of Veterinary Sciences of the University of Lodi (Italy), developed an experimental study to test a new robotic device: Telelap ALF‐x with the aim of evaluating the technical feasibility of the major surgical procedures “in vivo.” In 2015, the Italian SOFAR was acquired by the US company Trans‐Enterix.

The specific surgical function of the Telelap ALF‐x robot system is similar to the Da Vinci^**®**^ system, and thus competes in the market with it. The Telelap ALF‐x robotic system can monitor the doctor's eye, check the angle of the endoscope, and activate various surgical tools.^13^ In addition, the main characteristics of the system are the treatment of perception and value of force feedback, which allows the doctor to feel the force exerted by the surgical tool on the surgical tissue. It has a patented device for measuring the force applied by the surgical instrument to the surgical tissue. Its sensitivity is 0.35 N. This patent gives it a true sense of touch, which makes the operation safer and reliable.

Trans‐Enterix, in 2016, reintroduced The ALF‐X Robotic Surgical System As Senhance.

### 2.9 Hugo™ robotic‐assisted surgery system

The Hugo robotic‐assisted surgery (RAS) RAS system is a master–slave robotic platform designed for different procedures by Medtronic. The slave system is composed by surgical robotic dexterous arms with 7 DoF: each one, mounted on a single base, can carry wristed instruments specific for the surgical procedure. The master provides HD visualization of the site to the surgeon requiring 3‐D glasses and intuitive haptic interfaces.^14^ The system includes the cloud‐based surgical video capture option in Touch Surgery™ Enterprise with dedicated software modules to support robotics programme optimization, and training option. Hugo™ RAS system in October 2021 received CE (European Certification) Mark approval authorising the sale of the system in Europe for urologic and gynaecologic procedures.

### 2.10 Versius minimally invasive robotic system

Cambridge Medical Robotics Surgical, a UK company founded in 2014, realized Versius, a robotic system which seeks to allow robotic minimally invasive operations for a wide number of surgical procedures including gynaecological, urological, and colorectal surgical procedures. It is modularly conceived and consists of multiple identical arms, each mounted on a single support and taking small laparoscopic instruments (5 mm diameter). Each arm is very dexterous having 7 DoF. The master console includes a 3D‐HD imaging from the endoscopic camera with joystick controllers; haptic feedback is available.^15^

### 2.11 Ottava system by Johnson & Johnson

The Johnson & Johnson Ottava System is developed together with the company Verb Surgical (Santa Clara), founded in 2015.^16^ It will offer more flexibility and control than today's market systems mainly in the soft tissue robotic space. The new Ottava system has six arms that will be integrated into the operating table to provide greater control and flexibility in surgery.^17^ The platform has a zero‐impact design to allow patient access, increase Operating Room (OR) space and improve workflow. The company is now planning to begin the verification and validation processes for Ottava and is considering following enrolment in clinical trials for the device in the next years.

### 2.12 Bitrack surgical system

RobSurgical Systems, a spinoff of Universitat Politècnica de Catalunya (UPC), was created to exploit the Bitrack system, which is a laparoscopic surgical robot, designed at UPC.^18^ The Bitrack system, consists of four robotic arms mounted on a column through a prismatic joint that provides a linear DoF. Each arm can independently pivot around the column. The arms, with two differrent architectures, are placed in two levels. The two upper arms have a Selective Compliance Articulated Robot Arm architecture to avoid interferences with the two lower arms. The lower arms, having an antropomorphic architecture, are the operational arms. The kinematics of the system is studied in order to minimise the interferences between the arms. The redundancy of the system is set at the minimum (7 DoF) and, together with the collaborative control, is used to improve surgery procedures and to avoid collisions. Through a light open console, the surgeon can drive and control the procedures also supported by vision and haptic feedback functions. CE and FDA regulatory approvals are expected soon.

### 2.13 Enos single access robotic surgical system

The Enos surgical system is the single‐port orifice robotic technology surgical system proposed in 2020 from the Canadian company Titan Medical.

Enos is a master–slave robotic platform. The robot slave is able to move, elevate, tilt, and pan in a 25 mm insertion tube, two articulating flexible arms and two lighted camera systems; a 2D high‐definition camera and a 3D high‐definition camera that, under surgeon control, ensure seamless visibility of the surgical site.

Titan Medical's hyper‐redundant multi‐articulated instruments can position with dexterity the end effectors for grasping, suturing, cutting and coagulation. The open architecture anticipates future adaptability for new functionality and new end effectors. The master Enos workstation includes a 3D high‐definition display to provide surgical immersion and situational awareness in the OR. The handle interface is ergonomic and comfortable to surgeon.^19^ The small Enos footprint requires reduced hospitals surgical theatre area and lowers operating costs.

### 2.14 Hintori surgical robotic system

The Japanese companies, Kawasaki Heavy industries, a leading company of industrial robots, and Sysmex, an experienced business player in medical field, through a joint venture in 2013 established Medicaroid. Medicaroid started developing the Hinotori surgical robot system and relative working instruments and supports to medical staff including network services and IoT. The design of this surgical system is based on the concept of “co‐existence of humans and robot”.

It consists of three components: the Surgeon Cockpit, the Operation Unit, and the Vision Unit.

Arms of the Operation Unit are human inspired, designed to be as compact as human arms, which contributes to smoother operation because it reduces interferences between arms or between an arm and an assistant, are dexterous with 8 DoF and move smoothly like human arms.

The Surgeon Cockpit adopts ergonomic design to reduce the physical and stress burden on surgeons. It allows the surgeon to view and control the surgical site and instruments by operating it using hands and feet. The Vision Unit provides high‐definition 3D images on the stereoscopic viewer and supports smooth voice audio communication between surgeons and assistants.^20^ The Hinotori surgical system received Japanese regulatory approval in August 2020.

### 2.15 Symani micro‐surgical system

The Symani^**®**^ Surgical System is a flexible platform designed to facilitate surgical procedures across any anatomical region by the Italian company Medical Micro Instruments. It consists of two robotic arms that can be endowed with the proprietary, NanoWrist^**®**^ robotic micro instruments. The 7 DoF offered by the wrist enable the precision and control necessary for surgeons' easy manipulation to perform delicate procedures and sutures. The system features 7‐20x motion scaling with tremor filtration to address the demands and complexity of microsurgery and super microsurgery. This powerful combination allows surgeons to scale their hand movements while performing accurate surgery tasks.

The Symani Console is ergonomic and allows the surgeon to directly control the manipulators in the same manner as they would with manual instrumentation. The console can be used with a heads‐up 3D visualization system.^21^ Symani got the CE Mark in October 2020.

### 2.16 Avatera surgical system

In 2019, the German company Avatera Medical received EC approval for the components of the surgical robotic system developed with Leibnitz University. The system is very compact and consists of 4 robotic arms with 7 DoF for the agile handling of instruments including endoscope.

The company has developed a series of surgical instruments with a diameter reduced to just 5 mm dedicated to urological and gynaecological applications to ensure minimal invasiveness to the patient.

Very recently, with the acquisition of an academic spinoff FORWARDttc, the company is improving its surgical system by integrating experiences in image processing, artificial intelligence, VR and cloud computing.^22^ The market debut is nearby.

### 2.17 The MrBot equipment

MrBot, from Johns Hopkins University, is a remotely actuated robot for the access of the prostate gland. The robot is image guided by Magnetic Resonance Images (MRI). MrBot is designed to perform trans perineal needle insertion and percutaneous interventions such as biopsy, thermal ablations, or brachytherapy.^23^ Often, robots have a metal frame and are actuated by electric motors. Only the nonmagnetic and dielectric materials are ‘transparent’ to MRI; for this reason, MrBot is exclusively made of plastics, ceramics, and rubbers. Also, the electricity is forbidden; pneumatic actuation is performed by a new step motor,^24^ with control feedback given by a light‐based encoder. Accuracy of MrBot is high; MRI‐guided needle targeting experiments showed that the tip of the needle may be placed within 1 mm of a desired target selected in the image. Size of MrBot is small enough to fit into the MRI machine close to the legs of the patient.

### 2.18 The acubot and Revolving Needle Driver equipment

Acubot robot has the task of automating and improving the accuracy of the percutaneous nephrolithotomy. AcuBot has a 6 DoF serial link architecture and can be controlled locally or remotely. Time and accuracy of robotic tele‐operations are similar to locally aided robotic interventions. The robot stems from a collaboration between Guy's Hospital, London and Johns Hopkins University; the tip of AcuBot carries a Revolving Needle Driver (RND). Most needle robots orient a needle guide and insertion is then performed manually. Revolving Needle Driver end‐effector is a fully actuated driver for needle insertion, spinning, release, and force measurement. This is one of the most complex, feature rich needle driver reported.^25^ Like a drill, the RND can spin the needle while inserting it. Force sensors measure the interaction of the nozzle with the patient and the force of needle insertion. Force feedback may be used for tracking and following the patient respiratory motion.

### 2.19 Flex robotic system

Medrobotics is studying a very flexible kinetically distributed flexible system (“snake arm”) to allow doctors to operate with non‐linear tortuous pathways through a single access in the body. A key advantage is that the robot avoids the use of heart–lung machines required for open heart surgery (e.g. valve repair). Furthermore, this less invasive way of operating allows to improve patient recovery and reduces the risks associated with current procedures. The problems of positioning accuracy and the three‐dimensional view of the operational scenario must be still overcome.^26^

### 2.20 Mazor X stealth robotic system

Medtronics' new Mazor X Stealth robotic system for assisted spine surgery was introduced to the market in 2019, following Medtronics' acquisition of Mazor Robotics, a company expert in building guidance systems for spine and brain surgery with minimally invasive procedures. Mazor X Stealth consists of a robotic arm that guides spinal implants and instruments during the procedure and a surgical procedure planning software. The latest Mazor X Stealth Edition integrates the Medtronic stealth navigation technology with the O‐arm interoperable scanner in its robotic platform.

Future versions should include better compatibility of the robot with instruments, further developments of the navigation system and a revamped system of imaging to be integrated with existing planning and navigation software^27^; however, the specific details are not yet released.

### 2.21 Sensei X robotic catheter system

The Sensei X of Hansen Medical Inc. is a robotic cardiac catheter manipulation system. It consists of a master in a remote workstation who translates user movement, controlled by an electromechanical slave that contains an internal guide with 275° degrees range within an external guide capable of 90° span. The movement of each component is driven by traction wires via a remote joystick or buttons on the main console. Manipulation allows the freedom of movement and the ability to manoeuvre in three dimensions the catheter tool via remote control.^28^ Sensei X is equipped with a navigation system and with IntelliSense™ sensor system that allows quantification of the force applied by the catheter tip. All information is integrated and displayed visually on suitable monitors, including 3D mapping, ICE, fluoroscopy, and EKG (ElectroCardioGram) recordings.

### 2.22 Vascular catheter CorPath GRX

In 2019 Siemens Healthineers acquired Corindus Vascular Robotics, which developed a similar system, the CorPath GRX that enables precise trajectory control of coronary guide wires and balloon/stent devices. CorPath GRX helps improve workplace safety for interventional operators by allowing them to perform procedures from a radiation‐shielded workstation.^29^

### 2.23 Aeon Phocus cardiac catheter

A recent system is the Aeon Phocus developed by Aeon Scientific, a spin‐off from the ETH (Swiss Federal Institute of Technology) Zurich. It is a catheter guiding system for the treatment of cardiac arrhythmias. The patented technology allows, using magnetic fields generated by electromagnets, to remotely control interventional instruments such as catheters and guide wires in patients with great precision, thus reducing invasiveness and maximising efficacy, safety and efficiency.^30^ The University Medical Centre of Freiburg began distributing one of these intracardiac catheter devices in April 2021. The medical centre appreciates the high accuracy of the system and the improved safety for operators.

### 2.24 Viky endoscopic robot

The Viky from Endocontrol Medical is a lightweight endoscopic robot.^31^ Its role is to maintain and move the endoscope precisely according to the surgeon's orders while providing stable images, optimising the exposure of the surgical site. It is typically used for laparoscopy operations on the digestive, urological and gynaecological systems. The robot allows the surgeon to check its position directly without the help of an assistant.

### 2.25 ARES robotic endoscopy system

Auris Surgical Robotics, created Ares, a teleoperated endolumenal system designed to clarify the visualization of the respiratory tract during bronchoscopy.^32^ It consists of a slaver‐master system. The slaver (patient side) cart incorporates power box, control modules and a dual arms robot, each arm with 6 DoF and an instrumental driving mechanism with additional 4 active DoF. The master surgeon console, including haptic interaction with tactile force feedback, allows the surgeon to manipulate the instruments with amplified sensed forces and to perceive tissue consistency and the stress exerted by the instruments. The laparoscopic instruments are attached via magnets, facilitating their replacement and reuse.

Ares obtained the FDA certification for treatment and diagnosis of lung diseases. In 2019 Johnson & Johnson acquired the Auris Surgical Robotics company.

## 3 SMART TOOLS HELPING ROBOTIC SURGERY

Many researchers from industry and academia have developed and studied hardware and software devices to improve the performance of robotic surgical operations. Some of them are listed below.

### 3.1 The Freehand^®^ equipment

The Freehand^®^, by Prosurgics, is a remotely actuated laparoscopic camera controller launched in 2009. The surgeon, using a pedal and head movements, can control the scope position. Freehand^®^ is designed to cut the robot costs of the OR, while offering an instrument compact and easy to use.^33^ The equipment delivers steady images, during keyhole procedures. The surgeon needs to wear a head band to communicate with the robot. The hands‐free controller has the following working principle: first the surgeon, moving her/his head, chooses the scope tilt and pan direction, then the surgeon initiates the movement using the activation pedal. Remote camera zoom is also possible. The setup of the robot is quick: Freehand^®^ can be easily attached directly to the frame of the hospital beds.

### 3.2 The LAP Mentor™ equipment

The LAP Mentor™, from Simbionix™, is a training system in the field of laparoscopic surgery. The surgeon can try surgery procedures on a virtual patient. The human anatomy is rendered by simulation software; visualization is realistic. Virtual patients come from a 3D library of realistic anatomies created from images of real patients.^34^ The training system is modular: according to the simulation to perform, specific interchangeable handles can be used. Tactile feedback is also present. LAP Mentor™ Express is a cost‐effective portable version of the Lap Mentor™ platform; software runs on a laptop; the size of the virtual simulator is miniaturised. A wide range of surgical procedures modules is available. The surgeon can gain proficiency in inserting the needle, suturing and knotting using the Suturing Modules. Similarly other specific modules allow performing Lap Chole Procedural Tasks, Incisional hernia, Gastric bypass, Colorectal procedures and Salpingostomy procedures. Difficult and uncommon procedures may be practiced at any time with no risk.

### 3.3 The Axesse™ equipment

The Axesse™, by Elekta, is a 6 DoF intensity‐modulated stereotactic radiation system: the treatment is 3D image guided.^35^ Applications include stereotactic radiosurgery, stereotactic radiation therapy, stereotactic body radiation therapy and radiosurgery (stereotactic body radiation radiosurgery). The image system enables visualization of soft tissues; each time the patient makes a new session of therapy, the robot targets exactly the tissues interested. The patient is immobilised using vacuum to enable a better accuracy of the treatment. A 900 mm clearance around the patient improves the comfort during the treatment.

## 4 KEY ENABLING TECHNOLOGIES

Despite the great advantages that surgical robotics offers both to the patient, to the surgeon and to society, there are still limitations in the procedures due, above all, to the miniaturised mechatronic design, to the perceptual haptic interfaces, to the increase in autonomy that the development of new systems robotics aims to improve.

This section reports on the most relevant key technologies that underpin current and evolving developments, but it does not provide a complete overview of research topics in the field.

### 4.1 Mechanical design towards miniaturisation

The robotic instruments must be small in diameter to minimise the lesion of the patient, they must be stiff enough to allow for a sufficient positional accuracy and manipulation force; moreover, they must provide sufficient motion dexterity to reach the surgical site without damaging the organs and perform the surgical operation tasks there.

To meet these competing requirements, the miniaturisation of links, joints and end‐tools is needed including actuators^36^ and sensors.^37^ Dexterity can be obtained with a suitable number of actuated degrees of freedom, or with an arm with an underactuated and soft continuum structure to avoid harm to the patient.^38^ The slave robot design must be bio‐inspired^39^ and bio‐compatible^40^ with reference to the adoption of adequate structures and materials. Bio‐nano components will be integrated into surgical robotic structures and instruments^41^, ^42^ Soft robots, due to their versatility, have the potential to provide solutions for applications that rigid robots are not able to satisfactorily solve, for example, in the field of surgery where they will play a key role also for their inherent safety. The development of soft robotics is mainly fuelled by advances in three core areas: smart materials, mathematical modelling of compliant systems, and fabrication technologies.

### 4.2 Sensoring and perception

The adoption of robotic surgery systems introduces a new scenario in the operating theatre where the surgeon is separated from robot and patient and does not interact directly with the patient but through the remote‐control console.

It is necessary to make them virtually close and improve the haptic perception of the scenario by the surgeon with reflection of the forces exerted between robot and patient mainly during complex tissue operations, like ablation and suturing that sometimes require cooperation of the instrumental tools.^43^

The haptic feedback is a combination of kinaesthetic and tactile feedback. The first one measures the force that the system applies on the patient and return it to surgeon's hand via force feedback device. The second aims to create the perception that the surgeon's fingertip try out contacting the patient or surgical material.

Tactile sensors and haptics enabling force feedback with high resolution are designed by several researchers to provide the surgeon with a good sensitivity and immediacy of the in‐progress operation.^44^

Advanced vision systems are already available, but they continue to be improved with the aim of providing the surgeon with a clear view of the scenario to minimise the possibility of errors.^45^

The new artificial vision systems offer not only a 3D clear vision but, through advanced methods extending spatio‐temporal deep learning to 4D, provide the surgeon an in‐depth analysis of multi‐dimensional image representations of the intervention area, making the surgical operation more effective.

### 4.3 Teleoperation versus autonomy

Entrusting the slave operating tool to the robot, maximum transparency of the procedure in progress must be guaranteed to the master surgeon who operates remotely, making the interfaces simple, immediate, and intuitive.^46^

The surgeon acquires knowledge of the operative site and the progress of the surgical procedure through the interfaces which, by processing the information received from the multi‐sensory system in situ, offer her/him the realistic haptic perception of the situation on which to base the decisions for driving the intervention.

It is equally important that the decisions made by the surgeon are transmitted in an accurate, simple and effective way with immediacy to the robotic system that physically performs the tasks of the operation through suitable smart interfaces.

The teleoperation that takes place at a great distance, for example, when the surgeon is called to operate a patient who is on an offshore ship or on a naval or space platform or in a military field during a conflict, today can successfully exploit 5G and IoT technologies.

Today artificial intelligence is rapidly developing and becoming pervasive in a variety of sectors.

There are researchers who try to, at least partially, transfer the knowledge and methods of surgical intervention of surgeons to the robotic systems.^47^, ^48^

It is a challenge, that involves not only technical‐scientific problems, but also and above all ethical and acceptance problems.

Autonomous intelligent surgical robots will be proposed, and their performance will be tested in simple operations.^49^ It will be appropriate to grant procedures for the safe recovery and management of anomalies in the event of unexpected occurrences.^50^

### 4.4 Simulation for empowering precision care and training

Virtual surgery simulation systems that can reproduce the surgical system model and visualise the entire surgical process a priori on the computer are very useful for training the surgeons^51^ and to provide them a tool for improving the surgical procedures through a series of tests and trials to evaluate their efficiency and results.^52^

Surgical simulation mainly uses VR and augmented reality (AR) technologies applied to the hardware and software model of the robotic system to simulate and guide the various operating procedures.^53^ The purpose of the implementation includes planning the surgery with related tests, guidance during the surgery, the assistant's operation during the surgery, and sometimes suggestions for post‐operative rehabilitation.

Virtual reality, simulation and Digital Twin have the aim of replicating a real situation in a riskless environment, with lower costs. They are used both to train the abilities of trainees in any situation and to test a system before physically implement it.

Because the real robot system, before to be released to the market, is used in a real case intervention on phantom models, in vitro and in ex vivo cases, VR and digital twins are largely used. They are very promising for a refinement and improvement of both twins: the simulator and the surgical system.

Today, this kind of simulators has gradually become a new direction of research. It includes: robot kinematics, dynamic and visual models, 3D and AR representation of the surgical sites, modelling and simulation of the physical characteristics of the complex soft and hard objects like human organs present in the scenario, models and 3D image of navigation paths and assistance surgical techniques, simulation training modules.^54^

## 5 DISCUSSION

The robotic systems introduced are now compared: each system is classified by its spectrum of application and features (Table 1). A restricted range of features has been selected to compare the systems. Usually, the surgical systems take a large space of the surgical room: in case of emergency, the free access to the patient could be crucial. The doctor is no longer able to use his senses to directly interact with the patient: hence the intuitive representation of the surgery scenario and the haptic feedback become paramount. Also, some ‘physical’ features are introduced such as: modularity and reconfiguration of the system and surgical tools miniaturisation. The presented robotic systems are at different development stage: there are research projects under development, other systems under testing, and finally some robotic systems are commercially available on the market. The criterion introduced to produce this summary is merely subjective. On top of that, the scenario is rapidly changing; each robotic system is timely upgraded with enhanced features, and can be used for a wider spectrum of applications.

**TABLE 1:** Comparison of the robotic systems: spectrum of applications and features

Robotic system	Spectrum of applications	Features							
		Space occupied in the surgical room:	Accessibility to the patient in case of need	Effective and intuitive representation of the surgery scenario to the surgeon	Modularity and reconfiguration of the system	Surgical tools miniaturiz.	Haptic/force feedback	Certification	On the market
		L = large, M = medium, S = small	G = good, M = medium, L = limited	VG = very good, G = good, F = fair	G = good, M = medium, L = limited	S = standard, A = advanced	Y = yes, N = no	Y = yes, N = no	Y = yes, N = no
Zeus™	Thoracic,	L	L	F	L	S	N	Y	N
da Vinci®	Thoracic, urologic	L	L	VG	M	A	Under study	Y	Y
M7	Thoracic	S	G	G	L	S	Y	N	N
Raven IV	Thoracic	S	L	G	G	S	N	N	N
PMAR	Thoracic	S	L	G	L	S	N	N	N
Mirosurge	Thoracic	S	M	G	G	A	Y	N	N
MSR‐5000 REVO‐I	Thoracic urology	L	L	G	L	S	Y	Y	Y
ALF‐X	Thoracic, urology, gynaecol.	L	L	G	G	A	Y	Y	Y
Hugo	Urologic, gynaecol.	S	G	VG	M	A	Y	Y	Y
Versius	Thoracic, urologic, gynaecol.	S	M	G	G	A	Y	Y	Y
Ottava	Thoracic	S	M	G	G	S	‐	N	N
Bitrack	Thoracic, urology, gynaecol.,	L	L	G	M	S	Y	N	N
Enos	Urology, gynaecol.	S	G	G	M	S	N	Expected in 2022	
Hintori	Thoracic, urologic	L	L	VG	M	A	N	Y	Y
Symani	Open surgery	M	M	G	L	A	N	Y	Y
Avatera	Thoracic, urologic, gynaecol.	L	L	VG	M	A	Y	Y	Y
MrBot	Urologic	S	M	G	L	S	N	N	N
Acubot	Urologic	S	M	G	L	S	N	Y	Y
Flex	Transoral, urologic	S	G	G	M	S	N	Y	Y
Mazor X	Spine, brain	S	G	VG	M	‐	N	Y	Y
Sensei catheter	Endovasc.	S	G	VG	M	A	Y	Y	Y
GRX catheter	Endovasc.	S	G	G	M	A	Y	Y	Y
Aeon	Endovasc., cardiac	L	M	VG	L	A	N	Y	Y
Viky endosc.	Thoracic, urologic, gynaecol.,	S	G	G	L	S	N	Y	Y
Ares endosc.	Transoral	M	M	G	L	S	Y	Y	Y

## 6 CONCLUSION

The manuscript does not wish to be an exhaustive review on RASS; only a few robotic devices have been selected and described for each specific field of use. The objective of the work is to give a fresh feeling on how so many countries are simultaneously developing, and making available to the market advanced devices, that ultimately will contribute to extend life expectancy and improve the quality of human life.

The pioneers of the robotic surgery have built a strong reputation on the field, that now is used also by the newborn companies; the perception of the patients is radically changing from fear for the uncertain, to trust in a reliable technology tool. A rich history of successful clinical records helps the incoming patients to breathe a sigh of relief.

This ultimate wind of trust in this technology simultaneously influences both patients and companies. Venture capitalists, business angels and governments make funding available for the future development, because they think it is the right time for investments.

The technology race is starting now; while the first surgical robotics patents are expiring, a cloud of new patents are stemming to protect new promising ideas.

The sociopolitical factors also tend to promote advanced technology in the medical field. Governments continuously try to centralise the legislation; it is easier to penetrate a market where the same certification is available for a growing number of countries. For each specific medical procedure is necessary to obtain a Certificate of Clearance. The certification process takes time, effort and cost; a big ‘scale effect’ is obtained, in case the same procedure is automatically accepted on several countries.

The robotic surgery is already at an advanced level, with sophisticated solutions covering several areas where high accuracy, tremor filtering, force control, radiation protection, etc. suggest the interposition of a slave actuation, assuring the effectiveness and reliability to the remote handling under the surgeon's control.

During surgery tasks the maximum forces that need to be exerted by the end effectors are in the order of some hundred grams; it becomes clear that the next generation of surgical robot will be really compact.

Two different design methods may be applied, for the creation of the tomorrow medical robots; a conservative approach suggests the miniaturisation of the today medical devices, while a groundbreaking one may propose totally new robotic architectures specifically casted on the outcome that must be achieved; for example, the next generation of medical robots may derive from the further miniaturisation of serial robots, coming from the automation world, or may be form the ‘growing’ of bio‐nano robots.

The biggest research challenge is the simultaneous need for micro‐manipulation in local interventions, and raw motion at the handling level: this conflict may be resolved by the master/slave option, to perform micro‐manipulation by miniaturised slaves, scaled from natural size motion by the master controller. This architecture, however, needs specific information infrastructures, with focus on the direct and indirect potential of the effectors, and, also, to the opportunities offered by a computer integrated support. In general, the big gap between research and industry in surgical robotics can be filled with large investments in development, patents and advertisements. A warm wish is the next generation of robots to be lighter, more cost effective and therefore available to a wider public.

## CONFLICT OF INTEREST

The authors have declared no conflict of interest.

## Data Availability

Data sharing not applicable – no new data generated, or the article describes entirely theoretical research.

## References

[1] Bogue R . The rise of surgical robots. Ind Robot. 2021.

[2] Zhang X , Ma X , Zhou J , et al. Summary of medical robot technology development. IEEE ICMA. 2018:443‐448.

[3] Khandalavala K , Shimon T , Flores L , et al. Emerging surgical robotic technology: a progression toward microbots. Ann. Laparosc. Endosc. Surg. 2018(5):1‐18.

[4] Díaz CE , Fernández R , Armada M , et al. State of the art in robots used in minimally invasive surgeries. Natural orifice transluminal surgery (NOTES) as a particular case. Ind Robot. 2015.

[5] Díaz CE , Fernández R , Armada M , et al. A research review on clinical needs, technical requirements, and normativity in the design of surgical robots. Int. J. Med. Rob. Comp. Ass. Surg. 2017;13(4). 10.1002/rcs.1801 28105687

[6] Bucolo M , Buscarino A , Fortuna L , Gagliano S . Force feedback assistance in remote ultrasound scan procedures. Energies. 2020;13:3376.

[7] Bucolo M , Bucolo G , Buscarino A , et al. Remote ultrasound scan procedures with medical robots: towards new perspectives between medicine and engineering. Appl. Bionics Biomech. 2022. 10.1155/2022/1072642 PMC8832154 35154375

[8] Sumant U . Surgical robots market size, glob. Trends 2021–2027. https://www.gminsights.com/industry‐analysis/surgical‐robots‐market

[9] Marescaux J , Rubino F . The ZEUS robotic system: experimental and clinical applications. Surgical Clinics. 2003;83(6):1305‐1315. 14712867 10.1016/S0039-6109(03)00169-5

[10] Brodie A , Vasdev N . The future of robotic surgery. Ann R Coll. Surg. Eng. 2018;100(7):4‐13. 10.1308/rcsann.supp2.4 PMC6216754 30179048

[11] Rosen J , Lum M , Sinanan M , Hannaford B . Raven: developing a surgical robot from a concept to a transatlantic teleoperation experiment. Surg. Rob. 2011:159‐197.

[12] Seibold U , Kübler B , Bahls T , et al. The DLR MiroSurge surgical robotic demonstrator. Enc. Med. Rob. 2019(1):111‐142.

[13] Lococo A , Larocca V , Marino F , De Filippis AF , Cesario A , Lococo F . Experimental robotic pulmonary lobectomy with the TELELAP/ALFX system in the ovine model. Surg Innovat. 2015;22(3):252‐256. 10.1177/1553350614549633 25225214

[14] Millan B , Nagpal S , Ding M , Lee JY , Kapoor A . A scoping review of emerging and established surgical robotic platforms with applications in urologic surgery. Soc. Intern. d’Urologie J. 2021;2(5):300‐310.

[15] Morton J , Hardwick RH , Tilney HS , et al. Preclinical evaluation of the versius surgical system, a new robot‐assisted surgical device for use in minimal access general and colorectal procedures. Surg Endosc. 2021;35(5):2169‐2177. 32405893 10.1007/s00464-020-07622-4 PMC8057987

[16] Whooley S . Ottava surgical assistant robot finally unveiled by Johnson & Johnson. The Robot Report. 2020. Accessed: 2020‐12‐06. https://www.therobotreport.com/ottava‐surgical‐assistant‐robot‐finally‐unveiled‐by‐johnson‐johnson/.2020

[17] Klodmann J , Schlenk C , Hellings‐Kuß A , et al. An introduction to robotically assisted surgical systems: current developments and focus areas of research. Curr. Rob. Reports. 2021;2(3):321‐332.

[18] Casals Gelpi A , Frigola Bourlon M , Amat Girbau J . Bitrack: a friendly four arms robot for laparoscopic surgery. Proc. 10th Conf. New Tech. Comp./Rob. Ass. Surg. 2020:96‐97.

[19] Alip SL , Kim J , Rha KH , Han WK . Future platforms of robotic surgery. Urologic Clinics. 2022;49(1):23‐38. 34776052 10.1016/j.ucl.2021.07.008

[20] Bahreinian L . Humanizing the robot: medicaroid’s vision for the future of robotic surgery. Rob. Surg. 2021:165‐169.

[21] Ballestín A , Cuadros M . Microsurgery education in Spain. Issues of Reconstr. and Plast. Surg. 2021;24(1):97‐102.

[22] Liatsikos E , Tsaturyan A , Kyriazis I , et al. Market potentials of robotic systems in medical science: analysis of the Avatera robotic system. World J Urol. 2021:1‐7. 10.1007/s00345-021-03809-z PMC8381715 34424374

[23] Podder TK , Fenster A . Robotics in brachytherapy. Robotics Brachytherapy. Emerg. Tech. Brachyth. 2017:177‐196.

[24] Ludwig W , Badaan S , Stoianovici D . Robotic systems in urological surgery: current state and future directions. Rob. Genitourin surg. 2018:901‐908.

[25] Semins MJ , Stoianovici D , Matlaga BR . Percutaneous Nephrolithotomy Access: Robotic. Smith’s Textbook of Endour. 2019:269‐274.

[26] Manzo CE , Hartz KM , Walsh L , Lester C , Mathew A . Sa2062 transanal endoscopic submucosal dissection using the Flex® robotic system (medrobotics) by A gastroenterologist: a case series. Gastrointest Endosc. 2020;91(6):AB264‐AB265.

[27] O’Connor TE , O’Hehir MM , Khan A , et al. Mazor X Stealth robotic technology: a technical note. World neurosurg. 2021;145:435‐442. 33059080 10.1016/j.wneu.2020.10.010

[28] Russo AD , Fassini G , Conti S , et al. Analysis of catheter contact force during atrial fibrillation ablation using the robotic navigation system: results from a randomized study. J. Intervent. Cardiac Electrophys. 2016;46(2):97‐103. 10.1007/s10840-016-0102-0 26798037

[29] Britz GW , Panesar SS , Falb P , et al. Neuroendovascular‐specific engineering modifications to the CorPath GRX robotic system. J. neurosurg. 2019;133(6):1830‐1836. 10.3171/2019.9.JNS192113 31783367

[30] Hwang J , Kim JY , Choi H . A review of magnetic actuation systems and magnetically actuated guidewire‐and catheter‐based microrobots for vascular interventions. Intell. Serv. Rob. 2020;13(1):1‐14.

[31] Troccaz J , Dagnino G , Yang GZ . Frontiers of medical robotics: from concept to systems to clinical translation. Annu Rev Biomed Eng. 2019;21:193‐218. 30822100 10.1146/annurev-bioeng-060418-052502

[32] Peters BS , Armijo PR , Krause C , Choudhury SA , Oleynikov D . Review of emerging surgical robotic technology. Surg Endosc. 2018;32(4):1636‐1655. 29442240 10.1007/s00464-018-6079-2

[33] Anderson O , Arulampalam T . The FreeHand system. Handbook of Rob. and Image‐Guided Surg. 2020:57‐78.

[34] Oussi N , Enochsson L , Henningsohn L , Castegren M , Georgiou E , Kjellin A . Trainee performance after laparoscopic simulator training using a Blackbox versus LapMentor. J. Surg. Res. 2020;250:1‐11. 32014696 10.1016/j.jss.2019.12.039

[35] Zeng KL , Myrehaug S , Soliman H , et al. Stereotactic body radiotherapy for spinal metastases at the extreme ends of the spine: imaging‐based outcomes for cervical and sacral metastases. Neurosurgery. 2019;85(5):605‐612. 30169694 10.1093/neuros/nyy393

[36] Craker R , Johnson BV , Sakthivel H , et al. Design of a miniaturized actuation system for robotic lumbar discectomy tools. Intern. Design Eng. Tech. Conf. and Comp. and Inform. in Eng. Conf. 2020;83990:V010T10A041.

[37] Bandari N , Dargahi J , Packirisamy M . Miniaturized optical force sensor for minimally invasive surgery with learning‐based nonlinear calibration. IEEE Sensor J. 2019;20(7):3579‐3592.

[38] Gao RZ , Ren CL . Synergizing microfluidics with soft robotics: a perspective on miniaturization and future directions. Biomicrofluidics. 2021;15(1):011302. 33564346 10.1063/5.0036991 PMC7861881

[39] Stilli A , Althoefer K , Wurdemann HA . Soft robotics. Bio‐inspired antagonistic stiffening. Develop. Support Tech. 2018:207‐214.

[40] Halder A , Sun Y . Biocompatible propulsion for biomedical micro/nano robotics. Biosens. Bioelectr. 2019;139:111334. 10.1016/j.bios.2019.111334 31128479

[41] Soto F , Chrostowski R . Frontiers of medical micro/nanorobotics: in vivo applications and commercialization perspectives toward clinical uses. Frontiers in bioeng. and biotech. 2018;6:170. 10.3389/fbioe.2018.00170 PMC6246686 30488033

[42] Ummat A , Dubey A , Mavroidis C . Bionanorobotics: a field inspired by nature. Biomimetics Biol. Insp. Tech. 2005:201‐227.

[43] Mikada T , Kanno T , Kawase T , Miyazaki T , Kawashima K . Suturing support by human cooperative robot control using deep learning. IEEE Access. 2020;8:167739‐167746.

[44] Hamza‐Lup FG , Bergeron K , Newton D . Haptic systems in user interfaces: state of the art survey. Proc. Of the 2019 ACM Southeast Conf. 2019:141‐148.

[45] Fuerst B , Fer DM , Herrmann D , Kilroy PG . The vision of digital surgery. Digital Surg. 2021:11‐23.

[46] Li K , Ji S , Niu G , et al. Master‐slave control and evaluation of force sensing for robot‐assisted minimally invasive surgery. Ind Robot. 2020.

[47] Thai MT , Phan PT , Hoang TT , Wong S , Lovell NH , Do TN . Advanced intelligent systems for surgical robotics. Adv. Intell. Systems. 2020;2(8):1900138.

[48] Sandoval J , Laribi MA , Faure JP , Breque C , Richer J‐P , Zeghloul S . Towards an autonomous robot‐assistant for laparoscopy using exteroceptive sensors: feasibility study and implementation. IEEE Rob Autom Lett. 2021;6(4):6473‐6480.

[49] Attanasio A , Scaglioni B , De Momi E , Fiorini P , Valdastri P . Autonomy in surgical robotics. Annual Rev. of Control, Rob. and Auton. Systems. 2021;4:651‐679.

[50] Goldberg K . Robots and the return to collaborative intelligence. Nat Mach Intell. 2019;1(1):2‐4.

[51] Schlottmann F , Long JM , Brown S , Patti MG . Low confidence levels with the robotic platform among senior surgical residents: simulation training is needed. J. rob. Surg. 2019;13(1):155‐158. 10.1007/s11701-018-0853-y 30099663

[52] Rangarajan K , Davis H , Pucher PH . Systematic review of virtual haptics in surgical simulation: a valid educational tool? J. surg. educ. 2020;77(2):337‐347. 31564519 10.1016/j.jsurg.2019.09.006

[53] Hertz AM , George EI , Vaccaro CM . Head‐to‐head comparison of three virtual‐reality robotic surgery simulators. J Soc Laparoendosc Surg J. Soc. Laparoend. Surg. 2018;22(1). 10.4293/JSLS.2017.00081 PMC5863693 29618918

[54] Collins JW , Wisz P . Training in robotic surgery, replicating the airline industry. How far have we come? World J Urol. 2019:1‐7. 10.1007/s00345-019-02976-4 PMC7303079 31624867

